# Phase 1 study of the ATR inhibitor berzosertib (formerly M6620, VX-970) combined with gemcitabine ± cisplatin in patients with advanced solid tumours

**DOI:** 10.1038/s41416-021-01405-x

**Published:** 2021-05-26

**Authors:** Mark R. Middleton, Emma Dean, Thomas R. J. Evans, Geoffrey I. Shapiro, John Pollard, Bart S. Hendriks, Martin Falk, Ivan Diaz-Padilla, Ruth Plummer

**Affiliations:** 1 Department of Oncology, University of Oxford, Oxford, UK; 2 Experimental Cancer Medicine Team, The University of Manchester and The Christie NHS Foundation Trust, Manchester, UK; 3 Institute of Cancer Sciences, University of Glasgow and Beatson West of Scotland Cancer Centre, Glasgow, UK; 4 Department of Medical Oncology, Dana-Farber Cancer Institute and Harvard Medical School, Boston, MA USA; 5 Biological Sciences, Vertex Pharmaceuticals Europe Ltd, Abingdon, UK; 6 Clinical Pharmacology, EMD Serono Research & Development Institute Inc., Billerica, MA, USA, an affiliate of Merck KGaA, Darmstadt, Germany; 7 Oncology Global Clinical Development, Merck KGaA, Darmstadt, Germany; 8 Oncology Global Clinical Development, Ares Trading SA, Eysins, Switzerland, an affiliate of Merck KGaA, Darmstadt, Germany; 9 Translational and Clinical Research Institute, Newcastle University and Northern Centre for Cancer Care, Newcastle Hospitals NHS Trust, Newcastle Upon Tyne, UK; 10 Present Address: AstraZeneca, Cambridge and Alderley Park, UK; 11 Present Address: Bayer plc, Reading, UK; 12 Present Address: Novartis Institutes for BioMedical Research, Cambridge, MA USA; 13 Present Address: CureVac, Tübingen, Germany; 14 Present Address: GlaxoSmithKline, Zug, Switzerland

**Keywords:** Medical research, Cancer

## Abstract

**Background:**

Berzosertib (formerly M6620, VX-970) is a highly potent and selective, first-in-class inhibitor of ataxia telangiectasia and Rad3-related protein kinase (ATR). We assessed multiple ascending doses of berzosertib + gemcitabine ± cisplatin in patients with resistant/refractory advanced solid tumours.

**Methods:**

We evaluated the safety, tolerability, pharmacokinetics (PK) and preliminary efficacy of intravenous berzosertib + gemcitabine ± cisplatin using a standard 3 + 3 dose-escalation design. The starting doses were berzosertib 18 mg/m^2^, gemcitabine 875 mg/m^2^ and cisplatin 60 mg/m^2^.

**Results:**

Fifty-two patients received berzosertib + gemcitabine and eight received berzosertib + gemcitabine + cisplatin. Four patients receiving berzosertib + gemcitabine had a total of seven dose-limiting toxicities (DLTs) and three receiving berzosertib + gemcitabine + cisplatin had a total of three DLTs. Berzosertib 210 mg/m^2^ (days 2 and 9) + gemcitabine 1000 mg/m^2^ (days 1 and 8) Q3W was established as the recommended Phase 2 dose (RP2D); no RP2D was determined for berzosertib + gemcitabine + cisplatin. Neither gemcitabine nor cisplatin affected berzosertib PK. Most patients in both arms achieved a best response of either partial response or stable disease.

**Conclusions:**

Berzosertib + gemcitabine was well tolerated in patients with advanced solid tumours and showed preliminary efficacy signs.

**Clinical trial identifier:**

NCT02157792.

## Background

Chemotherapy drugs, such as platinum agents and gemcitabine, which induce potentially lethal DNA damage in cancer cells,^1^ are part of standard therapies for many cancers.^2–6^ Resistance to chemotherapy results in poor clinical outcomes. One mechanism implicated in both inherent and acquired resistance is the efficient repair of DNA damage in cancer cells through the activation of the complex DNA damage response (DDR).^7–9^

Apical regulators of DDR are ataxia–telangiectasia-mutated kinase (ATM) and ataxia telangiectasia and Rad3-related protein kinase (ATR), with ATR responding to exposed single-stranded DNA that often arises through DNA damage and replication stress, whereas ATM responds to DNA double-strand breaks. In preclinical studies, loss of ATM signalling (e.g., through ATM or p53 deficiency, or tumour protein [*TP53*] mutation) has been reported to drive reliance on ATR in response to DNA damage;^7,10–12^ subsequent loss of ATR signalling has been shown to result in synthetic lethality.^7^ ATR inhibition is an attractive therapeutic target for cancers in which DNA-damaging chemotherapy is utilised as standard therapy, but which retains a substantial unmet need.^13^

Berzosertib (formerly M6620, VX-970) is an intravenous (i.v.), highly potent and selective, first-in-class inhibitor of ATR (IC_50_ = 19 nM).^14,15^ Preclinical studies have demonstrated antitumour activity^15,16^ in synergy with chemotherapy, sensitising lung cancer cells to DNA-damaging agents, with the greatest effects observed with gemcitabine and cisplatin.^15^ In patient-derived lung tumour xenografts, berzosertib inhibited ATR activity and enhanced the efficacy of cisplatin, resulting in inhibition of tumour growth, including in tumours refractory to cisplatin monotherapy.^15^ Detailed in vitro and in vivo preclinical studies demonstrated that ATR inhibition was most effective when administered transiently, shortly after treatment with DNA-damaging chemotherapy. In mouse xenografts, a single berzosertib dose administered 12–24 h after chemotherapy was reported to be optimal and was superior to simultaneous administration.^17^

The purpose of this first-in-human, open-label, Phase 1 trial (ClinicalTrials.gov, identifier: NCT02157792) was to evaluate the safety, tolerability, pharmacokinetics (PK) and preliminary antitumour activity of berzosertib in combination with gemcitabine, with or without cisplatin.

## Methods

### Study design and treatment

**Fig. 1:**
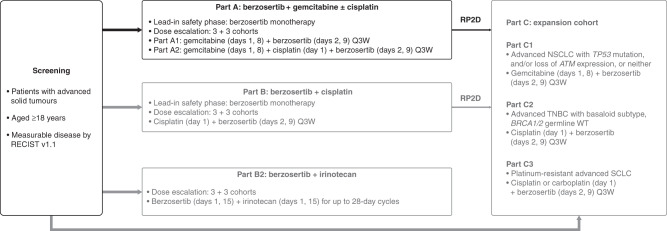
Study design (Part A reported here). ATM ataxia–telangiectasia-mutated kinase, DLX dose level X, NSCLC non-small-cell lung cancer, Q3W every 3 weeks, RECIST response evaluation criteria in solid tumours, SCLC small-cell lung cancer, TNBC triple-negative breast cancer, WT wild type.

This trial was part of a multicentre, open-label, non-randomised, Phase 1 study separated into six parts (A, B, B2, C1, C2 and C3). The focus of this paper is study part A: berzosertib in combination with gemcitabine with or without cisplatin in patients with advanced solid tumours; the other parts will be reported elsewhere. Part A was a 3 + 3 dose-escalation Phase 1 study evaluating the safety, tolerability, pharmacokinetics (PK) and preliminary efficacy of berzosertib in combination with gemcitabine with or without cisplatin (NCT02157792) (Fig. 1). Patients were enrolled in cohorts of three, with subsequent cohort expansion and dose-escalation decisions based on safety review, available PK data and observations of dose-limiting toxicities (DLTs) until the end of treatment cycle 1.

A 7–14-day lead-in period with single ascending doses of intravenous (i.v.) berzosertib was planned to assess the safety of berzosertib monotherapy; the requirement for the lead-in period was removed after review of safety data both during the study and from a concurrent study of berzosertib monotherapy.^17^ A 21-day chemotherapy dosing cycle was used for berzosertib + gemcitabine with or without cisplatin treatment. Based on preclinical studies, a starting dose of 18 mg/m^2^ and a dosing regimen of i.v. berzosertib on days 2 and 9, ~24 ± 4 h after dosing with gemcitabine ± cisplatin, was selected.^17^ For safety reasons, at study initiation and during the initial berzosertib dose-escalation phase, lower starting doses of gemcitabine (875 mg/m^2^) and cisplatin (60 mg/m^2^) were chosen than routinely used in the clinic.^18,19^ Patients received i.v. gemcitabine on days 1 and 8, with or without i.v. cisplatin on day 1. In the berzosertib + gemcitabine +  cisplatin cohorts, the starting dose of berzosertib did not exceed the highest dose of berzosertib utilised in the berzosertib + gemcitabine cohorts.

The berzosertib dose was escalated, while the dose of chemotherapy was kept constant until the berzosertib maximum tolerated dose (MTD) in combination with gemcitabine (875 mg/m^2^) ± cisplatin (60 mg/m^2^) was reached. The MTD was defined as the highest dose of berzosertib tolerated in combination with a cisplatin dose between 60 and 75 mg/m^2^ ± a gemcitabine dose between 500 and 1250 mg/m^2^, inclusive. At this point, doses of gemcitabine could be increased up to 1250 mg/m^2^ and cisplatin up to 75 mg/m^2^ in additional cohorts. In the presence of DLTs, the protocol allowed for reduction in gemcitabine and/or cisplatin dose to enable escalation of berzosertib in additional cohorts. Patients received treatment until progressive disease, unacceptable toxicity or withdrawal of consent.

### Patients

Eligible patients were ≥18 years of age with histologically or cytologically confirmed metastatic or unresectable solid tumours and measurable disease according to Response Evaluation Criteria in Solid Tumours (RECIST) version 1.1,^20^ for which standard curative or palliative therapy did not exist or was no longer effective at the time of study enrolment, or for whom treatment regimens containing gemcitabine and cisplatin might be considered. Patients for whom gemcitabine and/or cisplatin were appropriate treatment options were informed that, given the exploratory nature of this study, the doses of chemotherapy would be lower than those given in standard regimens, but were deemed clinically justified when administered in combination with berzosertib. Eligible patients had a World Health Organization performance status of 0–1, adequate bone marrow, liver and kidney function and a life expectancy of at least 12 weeks. In addition, patients were required to have received ≤6 cycles of prior chemotherapy with cisplatin and/or carboplatin (unless approved by the sponsor medical monitor).

Key exclusion criteria included radiotherapy (except palliative), endocrine therapy, immunotherapy or chemotherapy within the 4 weeks prior to receiving study therapy, more than six cycles of prior treatment with cisplatin, ongoing toxicity or recent major surgery (≤2 weeks of the first dose of study drug), active central nervous system disease or symptoms within 4 weeks prior to treatment, cardiac conditions within 6 months prior to treatment, prior bone marrow transplant or radiation to >15% of bone marrow and receiving medications that are known to be strong inhibitors or inducers of CYP3A4 that could not be discontinued at least one week before the start of treatment and for the duration of the study.

Full inclusion and exclusion criteria are shown in the Supplementary Information.

### Study assessments and endpoints

The primary objective of the study was to assess the safety and tolerability of multiple ascending doses of i.v. berzosertib, in combination with gemcitabine with or without cisplatin, in patients with advanced solid tumours. The secondary objectives were to determine the MTD, PK and preliminary antitumour activity of berzosertib in combination with cisplatin. Safety endpoints assessed were treatment-emergent AEs, the incidence of DLTs, clinical laboratory values, electrocardiogram outcomes and vital signs. Secondary endpoints were the MTD of berzosertib in combination with gemcitabine 500–1250 mg/m^2^ with or without cisplatin 60–75 mg/m^2^, PK parameters of berzosertib and the response of advanced solid tumours to berzosertib in combination with gemcitabine with or without cisplatin.

Treatment-emergent adverse events (TEAEs) and DLTs were reported using National Cancer Institute (NCI) Common Terminology Criteria for Adverse Events (CTCAE) version 4.0. The DLT assessment period was limited to cycle 1. All AEs of any grade were recorded and followed up until resolution. Safety was evaluated throughout treatment and was used to inform dose-escalation decisions. Dose-limiting toxicities (DLTs) were generally defined as any grade ≥3 haematologic or organ toxicity and any cardiac abnormality. Patients were eligible for DLT analysis if they either had a DLT before day 21 in cycle 1 or received all doses of berzosertib and gemcitabine with or without cisplatin in cycle 1 (see Supplementary Information for full definitions of DLTs).

PK assessments for berzosertib were conducted during the monotherapy lead-in and during cycles 1 and 2. Blood samples for berzosertib plasma PK assessments were collected following the single-agent dose, from day 1 of combination dosing (pre-dose, 0.5 h before the end of infusion, at end of infusion and 0.5, 1, 2, 3, 7, 23, 47 and 71 h after the end of infusion), on day 9 (pre-dose) and cycle 2 day 2 (pre-dose and 2 h after the end of infusion). Cumulative urine samples were collected for PK assessments on days 2 and 3 of cycle 1 (pre-dose—3, 3–7, 7–11 and 11–23 h). Berzosertib concentrations were quantified using a validated liquid chromatography tandem-mass spectrometry method and plasma PK was characterised by non-compartmental analyses using Phoenix WinNonlin 6.4 (Certara USA Inc, Princeton, NJ, USA).

Tumour responses were assessed every two cycles, using computed tomography, magnetic resonance imaging or bone scans as deemed appropriate by the investigators, until progression of disease; responses were assessed by the investigator according to RECIST version 1.1.

### Statistical analysis

Planned enrolment was ~60 patients. Sample size and power was based on a standard 3 + 3 dose-escalation rule using a binomial model. The maximally tolerated probability of toxicity associated with the dose selected by the standard 3 + 3 dose-escalation rule was calculated to range from ~17 to 26%, with an upper bound of 33%.

Safety data, baseline patient demographics and disease characteristics were summarised descriptively for the combination safety set—which included all enrolled patients who received at least one dose of study drug. PK data were collected in the PK analysis set, defined as all enrolled patients who received at least one dose of berzosertib and provided at least one measurable post-dose sample. Efficacy analyses were performed for the full analysis set, which included all enrolled patients who had a baseline scan, received one or more doses of study drug and had one or more post-baseline disease assessments. Standard non-compartmental methods were used to determine PK parameters.

## Results

### Patient demographics and disposition

Between December 2012 and June 2017, 60 patients were enrolled across five sites in the UK and USA. Patient demographics and disease characteristics are shown in Table 1. Few patients in the berzosertib + gemcitabine cohorts (four (8%)) and none in the berzosertib + gemcitabine + cisplatin cohorts had previously received gemcitabine. Similarly, few patients had a history of prior treatment with cisplatin (berzosertib + gemcitabine: ten (20%), berzosertib +  gemcitabine + cisplatin: two (25%), Table 2).

**Table 1:** Patient baseline demographics and characteristics (safety set).

	Berzosertib + gemcitabine (all doses), *n* = 50	Berzosertib + gemcitabine + cisplatin (all doses), *n* = 8
Sex, *n* (%)		
Male	28 (56.0)	4 (50.0)
Female	22 (44.0)	4 (50.0)
Race, *n* (%)		
White	49 (98.0)	8 (100.0)
Asian	1 (2.0)	0
Median (range) age, years	62 (28–79)	52 (26–71)
Age category, *n* (%)		
<65 years	33 (66.0)	6 (75.0)
≥65 years	17 (34.0)	2 (25.0)
Primary tumour, *n* (%)		
NSCLC	6 (12.0)	1 (12.5)
Pancreatic cancer	2 (4.0)	0
Breast cancer	4 (8.0)	0
Head and neck cancer	1 (2.0)	0
Colorectal cancer	18 (36.0)	4 (50.0)
Other^a^	19 (38.0)	3 (37.5)
WHO PS, *n* (%)		
0	15 (30.0)	3 (37.5)
1	35 (70.0)	5 (62.5)
Prior chemotherapy, *n* (%)	49 (98.0)	8 (100.0)
Platinum-based chemotherapy	44 (88.0)^b^	8 (100.0)^c^
Non-platinum-based chemotherapy	49 (98.0)^d^	8 (100.0)^e^

*WHO PS* World Health Organization performance status, *NSCLC* non-small-cell lung cancer.

^a^Other did not include small-cell lung cancer or ovarian cancer.

^b^Ten (20%) patients received prior cisplatin; best response of complete response (*n* = 1), partial response (*n* = 3), stable disease (*n* = 3), progressive disease (*n* = 2) and not available (*n* = 1).

^c^Two (25%) patients received prior cisplatin; best response of stable disease (*n* = 2).

^d^Four (8%) patients previously received gemcitabine; best response to gemcitabine of partial response (*n* = 1), stable disease (*n* = 1), progressive disease (*n* = 1) and not available (*n* = 1).

^e^No patients received prior gemcitabine.

**Table 2:** Overview of TEAEs and TEAEs occurring in >15% of patients by preferred term (combination safety set).

Patients, *n* (%)	Berzosertib + gemcitabine (all doses), *n* = 50		Berzosertib + gemcitabine + cisplatin (all doses), *n* = 8	
	Any grade	Grades 3–4^a^	Any grade	Grades 3–4^a^
*TEAEs*				
AEs	49 (98.0)	38 (76.0)	8 (100.0)	8 (100.0)
Serious AEs	24 (48.0)	13 (26.0)	5 (62.5)	5 (62.5)
*Treatment-related AEs*				
AEs	48 (96.0)	26 (52.0)	8 (100.0)	8 (100.0)
Serious AEs	14 (28.0)	4 (8.0)	4 (50.0)	4 (50.0)
AEs leading to study drug discontinuation	9 (18.0)	5 (10.0)	1 (12.5)	1 (12.5)
AEs leading to death	1 (2.0)^b^		0	
DLTs^c^	4 (8.0)		3 (37.5)	
TEAEs occurring in ≥15% of patients in either group	Any grade		Any grade	
Fatigue	32 (64.0)		8 (100.0)	
Nausea	31 (62.0)		7 (87.5)	
Anaemia	26 (52.0)		3 (37.5)	
ALT increased	25 (50.0)		3 (37.5)	
Vomiting	22 (44.0)		3 (37.5)	
AST increased	19 (38.0)		3 (37.5)	
Pyrexia	18 (36.0)		3 (37.5)	
Constipation	16 (32.0)		3 (37.5)	
Decreased appetite	16 (32.0)		2 (25.0)	
Diarrhoea	15 (30.0)		3 (37.5)	
Cough	15 (30.0)		1 (12.5)	
Neutropenia	14 (28.0)		5 (62.5)	
Headache	13 (26.0)		1 (12.5)	
Influenza-like illness	13 (26.0)		0	
Lower respiratory tract infection	13 (26.0)		0	
Lethargy	12 (24.0)		3 (37.5)	
Thrombocytopenia	12 (24.0)		3 (37.5)	
Blood alkaline phosphatase increased	11 (22.0)		2 (25.0)	
Dyspnoea	11 (22.0)		1 (12.5)	
Back pain	10 (20.0)		0	
Abdominal pain upper	7 (14.0)		2 (25.0)	
Dizziness	6 (12.0)		3 (37.5)	
Urinary tract infection	6 (12.0)		3 (37.5)	
Oedema peripheral	6 (12.0)		2 (25.0)	
Leukopenia	6 (12.0)		2 (25.0)	
Stomatitis	6 (12.0)		2 (25.0)	
Gamma-glutamyl transferase increased	4 (8.0)		2 (25.0)	
Myalgia	4 (8.0)		2 (25.0)	
Abdominal discomfort	2 (4.0)		2 (25.0)	
Grade ≥3 TEAEs^d^	Grade ≥3		Grade ≥3	
Neutropenia	8 (16.0)		5 (62.5)	
ALT increased	8 (16.0)		1 (12.5)	
Fatigue	8 (16.0)		1 (12.5)	
Thrombocytopenia	5 (10.0)		3 (37.5)	
Anaemia	5 (10.0)		1 (12.5)	

*AE* adverse event, *ALT* alanine aminotransferase, *AST* aspartate aminotransferase, *DLT* dose-limiting toxicity, *TEAE* treatment-emergent adverse event.

^a^No grade 5 AEs were observed.

^b^Patient had a reported serious AE of progression of non-small-cell lung cancer as the cause of death.

^c^In berzosertib + gemcitabine cohorts, the following DLTs were observed: increased grade 3 ALT and grade 3 fatigue (one patient; berzosertib 72 mg/m^2^ + gemcitabine 875 mg/m^2^), increased grade 3 AST (one patient; berzosertib 90 mg/m^2^ + gemcitabine 500 mg/m^2^), increased grade 3 ALT, grade 2 AST, and grade 2 blood alkaline. phosphatase (one patient; berzosertib 140 mg/m^2^ + gemcitabine 500 mg/m^2^) and grade 4 thrombocytopenia (one patient; berzosertib 72 mg/m^2^ + gemcitabine 875 mg/m^2^). In berzosertib + gemcitabine + cisplatin cohorts, two patients in the berzosertib 120 mg/m^2^ cohort had DLTs (grade 4 febrile neutropenia and neutropenia); a third patient (berzosertib 90 mg/m^2^) had a DLT of thrombocytopenia (grade 4). All these patients also received gemcitabine 875 mg/m^2^ and cisplatin 60 mg/m^2^.

^d^Occurring in ≥10% of patients in the berzosertib + gemcitabine cohorts or in more than one patient in the berzosertib +  gemcitabine + cisplatin cohorts.

**Fig. 2:**
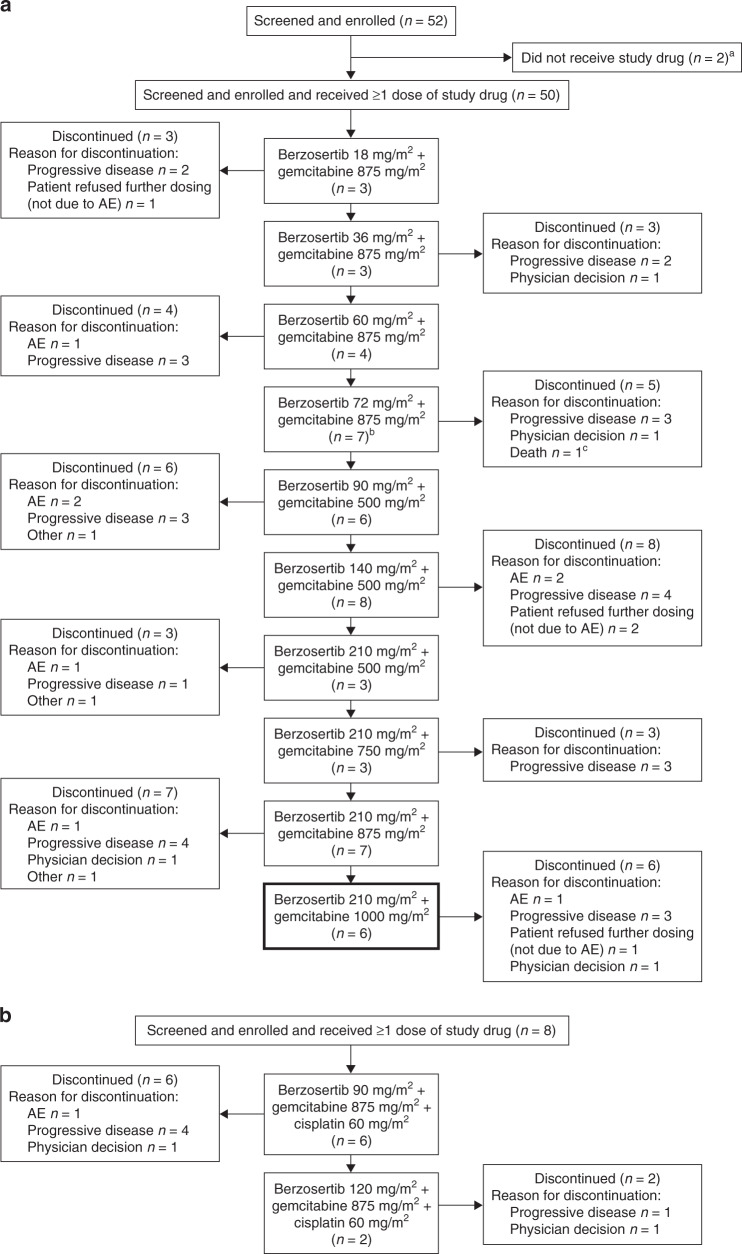
Patient disposition (all patients set). **a** Berzosertib + gemcitabine cohorts. **b** Berzosertib + gemcitabine + cisplatin cohorts. ^a^Two patients in the berzosertib + gemcitabine group (one patient each due to receive berzosertib 210 mg/m^2^ + gemcitabine 500 mg/m^2^, berzosertib 210 mg/m^2^ + gemcitabine 750 mg/m^2^) never received study treatment due to renal failure before or on day 1. ^b^Two patients in the berzosertib 72 mg/m^2^ + gemcitabine 875 mg/m^2^ cohort completed treatment per the protocol version at the time, which limited treatment to six cycles. A later protocol amendment eliminated the restriction on duration of treatment, and thereafter no patient was considered to have completed treatment. ^c^This patient had an AE leading to berzosertib discontinuation, however, the primary reason for discontinuation was recorded as death by the investigator. *Note*: intra-patient dose escalation did not occur. For berzosertib in combination with gemcitabine, berzosertib 210 mg/m^2^ + gemcitabine 1000 mg/m^2^ (bold outline) was the recommended Phase 2 dose. AE adverse event.

Patient disposition for the combination treatment period for berzosertib + gemcitabine with or without cisplatin is shown in Fig. 2. A total of 52 patients were enrolled over ten dose cohorts of berzosertib + gemcitabine, and 8 patients were enrolled across two dose cohorts of berzosertib + gemcitabine + cisplatin. The majority of patients discontinued study treatments due to progressive disease (berzosertib + gemcitabine, 58%; berzosertib + gemcitabine + cisplatin, 63%).

The median number of treatment cycles of berzosertib during the berzosertib + gemcitabine combination period was 4.0 (range: 1–20), and within each cohort, the mean relative dose intensity of berzosertib was 89.6%. The median duration of treatment (overall) in the berzosertib + gemcitabine combination period was 83.0 days (range: 2–430). In patients receiving berzosertib + gemcitabine + cisplatin, the median number of treatment cycles was 5.0 (range: 1–14) for both berzosertib + gemcitabine, and 3.5 for cisplatin (range: 1–7), with a mean relative dose intensity of berzosertib of 80.92%. The median duration of treatment (overall) for the berzosertib + gemcitabine + cisplatin combination period was 89.5 days (range: 2–290).

### Dose escalation and dose-limiting toxicities

In the berzosertib + gemcitabine cohorts, four (8.9%) patients had a total of seven DLTs; these were distributed across the different dosing cohorts as follows: berzosertib 72 mg/m^2^ + gemcitabine 875 mg/m^2^ = three DLTs in two patients, berzosertib 90 mg/m^2^ + gemcitabine 500 mg/m^2^ = one DLT in one patient and berzosertib 140 mg/m^2^ + gemcitabine 500 mg/m^2^ = three DLTs in one patient. No DLTs occurred in the lowest berzosertib dose groups (18–60 mg/m^2^). Of the three DLTs occurring in the two patients in the berzosertib 72 mg/m^2^ + gemcitabine 875 mg/m^2^ cohort, one patient had two DLTs (grade 3-increased ALT and grade 3 fatigue) and another patient had a DLT of grade 4 thrombocytopenia. Given these toxicities, for the next dose-escalation cohort, berzosertib was increased to 90 mg/m^2^ but gemcitabine was decreased to 500 mg/m^2^. The one patient in this cohort with a DLT experienced grade 3-increased aspartate aminotransferase (AST). Further dose escalation to berzosertib 140 mg/m^2^ + gemcitabine 500 mg/m^2^ resulted in one patient experiencing three DLTs (grade 3 ALT increase, grade 2 AST increase and grade 2 blood alkaline phosphatase increase). None of the liver function test elevations that were considered to be DLTs, were transient, and based on review of the available safety data, the DLT definition was modified to exclude transient grade 3 liver function test elevations (see Supplementary Information).

Subsequently, the berzosertib dose was increased to 210 mg/m^2^, initially in combination with gemcitabine 500 mg/m^2^, and then in three subsequent cohorts with gemcitabine 750, 875 and 1000 mg/m^2^. No DLTs were reported for these dose combinations. Gemcitabine dose escalation was stopped at 1000 mg/m^2^ due to emerging cumulative haematologic toxicity in later treatment cycles;^21^ in the berzosertib 210 mg/m^2^ + gemcitabine 1000 mg/m^2^ cohort, five patients presented with grade 1–2 anaemia, four with neutropenia (two each with grade 1–2 and grade 3) and three with thrombocytopenia (one each with grade 1–2, grade 3 and grade 4). Although the MTD for berzosertib in combination with gemcitabine was not reached, berzosertib 210 mg/m^2^ (days 2 and 9) + gemcitabine 1000 mg/m^2^ (days 1 and 8) every 3 weeks (Q3W) was chosen as the RP2D as the combination was well tolerated prior to the emergence of haematologic toxicity and the berzosertib dose of 210 mg/m^2^ exceeded that of the predicted efficacious dose based on preclinical studies.

In the berzosertib + gemcitabine + cisplatin cohorts, three patients had a total of three DLTs. One patient in the berzosertib 90 mg/m^2^ + gemcitabine 875 mg/m^2^ + cisplatin 60 mg/m^2^ cohort had a DLT of grade 4 thrombocytopenia; berzosertib was then escalated to 120 mg/m^2^ with the same doses of gemcitabine and cisplatin. Two patients in the berzosertib 120 mg/m^2^ cohort had DLTs, one grade 4 febrile neutropenia and one grade 4 neutropenia; both patients discontinued treatment. Following the observation of these DLTs, dose escalation was halted and the RP2D was not established for this combination therapy.

### Safety

Overall, 58 patients were included in the safety set; a summary of AEs for berzosertib + gemcitabine with or without cisplatin is shown in Table 2; fatigue and nausea were the most commonly reported TEAEs. Twenty-four patients (48%) who received berzosertib + gemcitabine experienced one or more serious AEs (SAEs), the most common of which was pyrexia, which occurred in six patients (12%), with all other SAEs occurring in two or fewer patients. In the berzosertib +  gemcitabine + cisplatin cohorts, nine patients (18%) experienced TEAEs resulting in discontinuation—the most common of which was fatigue (6%). Fourteen patients (28%) experienced treatment-related SAEs, the most common also being pyrexia (12%).

In patients receiving berzosertib + gemcitabine, grade ≥3 TEAEs of neutropenia, increased ALT and fatigue each occurred in eight patients (16%), with anaemia and thrombocytopenia each occurring in five patients (10%). The most common grade ≥3 TEAEs experienced by patients receiving berzosertib + gemcitabine + cisplatin were neutropenia in five patients (63%) and thrombocytopenia in three patients (38%). There were no deaths due to AEs in patients receiving berzosertib + gemcitabine + cisplatin. A full description of the number of treatment-related AEs of grade ≥3 by dosing cohort can be found in Supplementary Table S1.

Finally, there were no clinically meaningful trends attributable to berzosertib treatment identified from laboratory results (serum chemistry, haematology or urinalysis), vital signs or ECG parameters.

### Pharmacokinetics

**Fig. 3:**
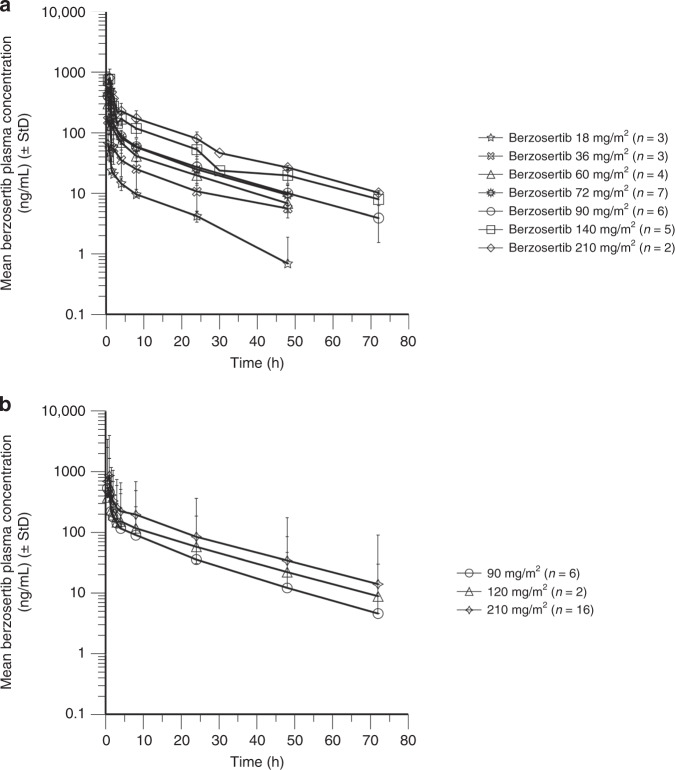
Berzosertib plasma concentration–time profiles (PK analysis set). **a** Single ascending doses of berzosertib for the lead-in period (berzosertib monotherapy). **b** Berzosertib + gemcitabine and berzosertib + gemcitabine + cisplatin cohorts. StD standard deviation.

Mean PK profiles for the monotherapy lead-in and combination with gemcitabine with or without cisplatin are shown in Fig. 3. PK parameters for berzosertib monotherapy lead-in were determined for 30 patients across the dose range of 18–210 mg/m^2^ and are shown in Supplementary Table S2. The plasma PK of berzosertib was characterised by biphasic decline with a moderate-to-high clearance, high distribution volume and apparent terminal half-life of approximately 17 h. Exposure was approximately dose-proportional from 18 to 210 mg/m^2^. Based on 16 patients for the combination of berzosertib 210 mg/m^2^ with gemcitabine and eight patients for the combination of berzosertib 90–120 mg/m^2^ with gemcitabine and cisplatin, berzosertib exposures (maximum observed concentration (C_max_) and area under the concentration vs. time curve (AUC_0-∞_)) were consistent with those for corresponding doses of berzosertib alone (Supplementary Table S2). For part A, the mean renal clearance of berzosertib was 3.6 L/h and the mean percentage of berzosertib excreted in the urine was 5%.

### Efficacy

**Fig. 4:**
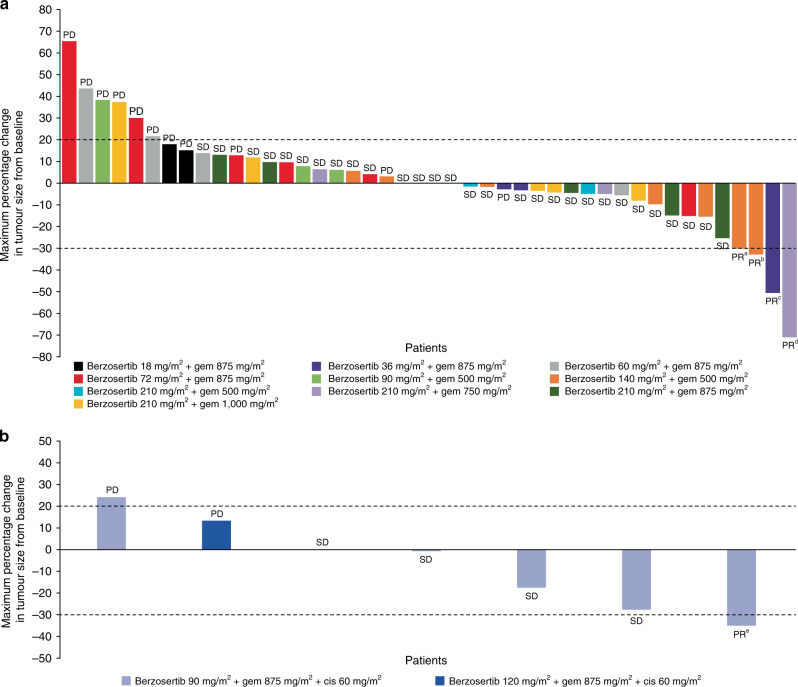
Tumour response. Maximum percentage change in tumour size from baseline and best overall response is shown for patients (full analysis set) receiving **a** berzosertib + gemcitabine (*n* = 44) and **b** berzosertib + gemcitabine + cisplatin (*n* = 7). The dashed line at 20% represents PD, whereas the dashed line at −30% represents PR. Patients with PR had the following primary tumour types: ^a^non-small-cell lung cancer; ^b^carcinoma (unknown primary origin); ^c^head and neck cancer; ^d^BRCA2 mutation-positive breast cancer; ^e^colorectal cancer. cis cisplatin, gem gemcitabine, PD progressive disease, PR partial response, SD stable disease.

Of the 48 efficacy-evaluable patients who received berzosertib + gemcitabine, 4 (8.3%) patients achieved a best response of partial response (PR) and 29 (60.4%) patients had stable disease (SD) as their best response (Fig. 4a). Of the four patients in the berzosertib + gemcitabine cohort who achieved a best response of PR, none had previously received gemcitabine, two had previously received cisplatin and the duration of response ranged between 71 and 211 days. One of the above patients, presenting with oestrogen and progesterone receptor-positive, HER2-negative, germline *BRCA2* mutation-positive metastatic breast cancer, achieved a confirmed PR after four cycles of berzosertib 210 mg/m^2^ + gemcitabine 750 mg/m^2^. She had previously experienced disease progression after carboplatin and PARP inhibitor (olaparib) treatment for metastatic disease. She completed 14 cycles of berzosertib + gemcitabine and discontinued study treatment due to progressive disease.^21^ Another patient, presenting with non-squamous, non-small-cell lung cancer (NSCLC, adenocarcinoma), achieved a best response of PR after 12 cycles of berzosertib 140 mg/m^2^ + gemcitabine 500 mg/m^2^; PR was sustained through follow-up week eight. With prior regimens, which included cisplatin, pemetrexed, erlotinib and docetaxel, he had only attained the best response of the stable disease.

In the seven efficacy-evaluable patients who received berzosertib + gemcitabine + cisplatin, one (14%) patient achieved a best response of PR and four (57%) patients had SD as their best response (Fig. 4b). The patient with a best response of PR had colorectal cancer and had not been previously treated with either gemcitabine or cisplatin. The duration of response for this patient was 94 days, following receipt of berzosertib 90 mg/m^2^ + gemcitabine 875 mg/m^2^ + cisplatin 60 mg/m^2^.

## Discussion

This was a first-in-human study exploring the safety, tolerability and PK of the first-in-class ATR inhibitor berzosertib in combination with gemcitabine with or without cisplatin in patients with advanced solid tumours who were resistant to standard therapy. The MTD of berzosertib with gemcitabine was not reached because target engagement was predicted at the highest berzosertib dose evaluated (210 mg/m^2^) and gemcitabine dose escalation was stopped due to emerging cumulative haematologic toxicity. Based on the tolerability of the combination, berzosertib 210 mg/m^2^ (days 2 and 9) +  gemcitabine 1000 mg/m^2^ (days 1 and 8) Q3W was established as the RP2D and was carried forward to be evaluated in patients with advanced NSCLC in an expansion arm of this study (part C1). A RP2D was not determined for berzosertib in combination with gemcitabine and cisplatin as berzosertib dose escalation was not continued after two patients experienced DLTs of febrile neutropenia or neutropenia in the berzosertib 120 mg/m^2^ + gemcitabine 875 mg/m^2^ + cisplatin 60 mg/m^2^ cohort.

Based on the safety profile reported, berzosertib was considered well tolerated only in combination with gemcitabine without cisplatin, however, all side effects reported were consistent with those reported for gemcitabine and cisplatin when administered as a single agent.^22^ However, the prevalence and/or severity of the observed AEs necessitated lower doses of gemcitabine or cisplatin than those administered as part of standard chemotherapy combination regimens.^17,23^

The PK of berzosertib when administered ~24 h after gemcitabine, with or without cisplatin, was not significantly different than that of berzosertib alone. Accumulation with weekly administration of berzosertib was not observed. As there was no reason to expect a PK interaction, the exposure of gemcitabine, with or without cisplatin, was not assessed in this study.

A total of five patients achieved PR across both cohorts, providing preliminary evidence of antitumour activity for the combination of berzosertib + gemcitabine with or without cisplatin in patients with advanced solid tumours. Further, all five patients with PR had previously received platinum-based chemotherapy, with three having received prior cisplatin or carboplatin. The response observed in a heavily pre-treated patient with advanced *BRCA2-*positive breast cancer who previously progressed on platinum chemotherapy and olaparib suggests that berzosertib may have a role in overcoming platinum and/or PARP inhibitor resistance, as has been demonstrated in preclinical experiments.^24^ The expansion cohorts (part C) of this study further evaluated berzosertib in combination with gemcitabine or cisplatin/carboplatin in tumour types in which *TP53* mutations and/or ATM deficiency are common, or which are likely to be under replicative stress—including NSCLC,^25^ triple-negative breast cancer^26^ and small-cell lung cancer^27^—and may be particularly susceptible to ATR inhibition.

Recently, berzosertib has been shown to be both well tolerated and efficacious in combination with gemcitabine in a Phase 2 randomised study (berzosertib + gemcitabine vs. gemcitabine alone) in platinum-resistant high-grade serous ovarian cancer (*n* = 70).^28^ It has also shown preliminary clinical antitumour activity in combination with other chemotherapy agents, including with topotecan in heavily pre-treated patients with advanced solid tumours, including durable responses in relapsed platinum-resistant small-cell lung cancer.^23^ A proof-of-concept Phase 2 study with berzosertib in combination with topotecan in patients with SCLC reported an objective response rate of 36% (9/25), with a duration of response ≥6 months.^29^ In a separate Phase 1 dose-escalation study, berzosertib has also been evaluated in combination with carboplatin, where it has shown some preliminary signs of activity in platinum and PARP inhibitor pre-treated patients.^17^ The combination of carboplatin and berzosertib is now being compared to carboplatin–docetaxel in men with pre-treated metastatic castrate-resistant prostate cancer (NCT03517969).

Future work should continue to evaluate potential biomarkers that may be predictive of response, in order to elucidate mechanisms of action and identify patients most likely to benefit from ATR inhibition in combination with chemotherapy. Other studies of berzosertib are currently investigating patient populations with advanced solid tumours selected by various genetic abnormalities, including *ATM* truncating mutations, germline *BRCA* mutation and other alterations likely to disrupt homologous recombination repair or cause replicative stress (NCT03718091).

In conclusion, this study has demonstrated that berzosertib is well tolerated in combination with gemcitabine in patients with advanced solid tumours. Berzosertib has also demonstrated preliminary clinical activity, building the foundation for subsequent Phase 2 studies, where these early signs of clinical activity have been confirmed.^28,29^ Further late-stage clinical evaluation of berzosertib is warranted and the identification of predictive biomarkers of response is the critical next step in order for berzosertib to potentially become an additional therapeutic option in the treatment armamentarium for cancer patients.

## Supplementary information

Supplementary Material

## Data Availability

Any requests for data by qualified scientific and medical researchers for legitimate research purposes will be subject to the Merck KGaA, Darmstadt, Germany Data Sharing Policy. All requests should be submitted in writing to the Merck KGaA, Darmstadt, Germany data-sharing portal (https://www.merckgroup.com/en/research/our-approach-to-research-and-development/healthcare/clinicaltrials/commitment-responsible-data-sharing.html). When Merck KGaA, Darmstadt, Germany has a co-research, co-development or co-marketing or co-promotion agreement, or when the product has been outlicensed, the responsibility for disclosure might be dependent on the agreement between parties. Under these circumstances, Merck KGaA, Darmstadt, Germany, will endeavour to gain agreement to share data in response to requests.
