# Supplementary material for: Phase 1 study of the ATR inhibitor berzosertib (formerly M6620, VX-970) combined with gemcitabine ± cisplatin in patients with advanced solid tumours
Source: Br J Cancer. 2021 May 26;125(4):510–9. doi: 10.1038/s41416-021-01405-x (PMC8368196; doi:10.1038/s41416-021-01405-x)
Supplement: Supplementary file 1 — Supplementary Material [file 41416_2021_1405_MOESM1_ESM.docx]

**Supplementary information**

# Full inclusion and exclusion criteria

## Inclusion criteria

Patients who met all the following inclusion criteria were eligible for this study:

- Male and female patients ≥18 years of age.
- Disease status: Patient with histologically or cytologically confirmed advanced solid tumour that was metastatic or unresectable and for which standard curative or palliative measures did not exist or were no longer effective, or for whom regimens containing gemcitabine, cisplatin, and/or etoposide might be considered, and with measurable disease according to Response Evaluation Criteria in Solid Tumors criteria (version 1.1).
- World Health Organization performance status of 0 or 1.
- Life expectancy of ≥12 weeks.
- Haematological and biochemical indices within the ranges shown below at screening, with no clinically significant change in these values confirmed on the first day of dosing, before study drug administration.
  - Haemoglobin: ≥8.0 g/dL; absolute neutrophil count: ≥1.5 × 10^9^/L; platelet count: ≥100 × 10^9^/L; serum bilirubin: ≤1.5 × upper limit of normal (ULN), unless the patient had known or suspected Gilbert’s syndrome; alanine aminotransferase (ALT) and aspartate aminotransferase (AST): ≤2.5 × ULN or ≤5 × ULN in presence of liver metastases; estimated glomerular filtration rate: ≥50 mL/min or ≥60 mL/min for patients planned to receive a cisplatin-containing regimen; prothrombin time: <1.5 × ULN. In addition, there were no other clinically significant metabolic or haematologic abnormalities that were uncorrectable or that required ongoing, recurrent pharmacologic management.
- Signed and dated an informed consent document.
- Was willing and able to comply with scheduled visits, treatment plan, lifestyle, laboratory tests, contraceptive guidelines, and other study procedures.

## Exclusion criteria

Patients who met any of the following exclusion criteria were not eligible for this study:

- Radiotherapy (except for palliative reasons), endocrine therapy, immunotherapy, or chemotherapy during the previous 4 weeks (6 weeks for nitrosoureas and Mitomycin-C, and 4 weeks for investigational medicinal products) or less than four drug half-lives, whichever is greater, before first dose of study drug.
- Prior chemotherapy: greater than 6 cycles of prior treatment with cisplatin and/or carboplatin, unless discussed with and approved by Vertex Pharmaceuticals (Vertex) medical monitor (history of prior dose reductions or dose interruptions while receiving cisplatin or carboplatin due to toxicity from the platinum or intolerance to either agent, unless discussed with and approved by Vertex medical monitor); known history of grade 4 thrombocytopenia or grade 4 neutropenia while receiving prior therapy, unless discussed with and approved by Vertex medical monitor.
- Unresolved toxicity of Common Terminology Criteria for Adverse Events (CTCAE) grade 2 or greater from previous anti-cancer therapy or radiotherapy, excluding: alopecia; anaemia or leukopenia, as long as screening haemoglobin and absolute neutrophil counts fell within limits specified in inclusion criteria 5a and 5b; other toxicities that in the opinion of the investigator and the sponsor should not exclude the patient.
- History of spinal cord compression or brain metastases, unless asymptomatic, treated, stable, and not requiring treatment with steroids for at least 4 weeks before first dose of study drug. Any history of leptomeningeal metastases.
- Women who were already pregnant or lactating or planned to become pregnant within 6 months of the last dose of study drug were excluded. Women of childbearing potential were required to adhere to contraception guidelines as outlined in the protocol. Women were considered to be of non-childbearing potential if they underwent surgical hysterectomy or bilateral oophorectomy or were amenorrhoeic for over 2 years with a screening serum follicle-stimulating hormone level within the laboratory’s reference range for postmenopausal females.
- Men with partners of childbearing potential were required to adhere to contraception guidelines in the protocol. Men with pregnant or lactating partners or partners who planned to become pregnant during the study or within 6 months of the last dose of study drug were excluded.
- Major surgery ≤2 weeks before starting study drug, or incomplete recovery from a prior major surgical procedure.
- Cardiac conditions as follows: Clinically significant cardiovascular event within 6 months before study entry to include: congestive heart failure requiring therapy; unstable angina pectoris; myocardial infarction; class II/III/IV cardiac disease (New York Heart Association); presence of severe valvular heart disease; presence of a ventricular arrhythmia requiring treatment; history of arrhythmia that is symptomatic or requires treatment (CTCAE 3), symptomatic or uncontrolled atrial fibrillation despite treatment, or asymptomatic sustained ventricular tachycardia (patients with atrial fibrillation controlled by medication were permitted); uncontrolled hypertension (blood pressure ≥160/100 despite optimal therapy); second- or third-degree heart block with or without symptoms; QTc >450 msec (by Fridericia’s correction) not due to electrolyte abnormality and that did not resolve with correction of electrolytes; history of congenital long QT syndrome; history of torsades de pointes (or any concurrent medication with a known risk of inducing torsades de pointes); clinically significant abnormality, including ejection fraction (EF) below normal institutional limits, present on transthoracic echocardiogram performed at screening.
- Prior bone marrow transplant or extensive radiotherapy to greater than 15% of bone marrow.
- Participation or plan of participation in another interventional clinical study while taking part in this phase 1 study of berzosertib. Participation in an observational study was acceptable.
- Any other condition that, in the investigator’s opinion, did not make the patient a good candidate for the clinical study, including: history of human immunodeficiency virus 1 (HIV-1), HIV-2, hepatitis C virus, or unresolved hepatitis B infection; high medical risk because of non-malignant systemic disease, including active uncontrolled infection; history of serious drug allergy or auto-immune disease; diagnosis of Li-Fraumeni Syndrome or ataxia telangiectasia.
- Patient was the investigator or a sub-investigator, research assistant, pharmacist, study coordinator, other staff, or a relative of study personnel directly involved with the conduct of the study.
- Current therapy: patients receiving treatment with medications that are known to be strong inhibitors or inducers of CYP3A4 that could not be discontinued at least a week before start of treatment and for the duration of the study. For patients who were to receive cisplatin in this study: patients receiving treatment with ototoxic or nephrotoxic medications that could not be discontinued at least 7 days before first dose of study drug and for the duration of the study. Inadvertent or short-term use on study did not cause a patient to be ineligible. If a short course of therapy with nephrotoxic or ototoxic medication was anticipated and required, cisplatin was discontinued until 7 days after this course was completed.

# Definition of dose-limiting toxicity (DLT)

DLTs were identified throughout the dosing cycles and defined using the National Cancer Institute (NCI) CTCAE (version 4.0). A DLT was defined as any of the following events that were considered related or possibly related to study drug:

- Neutropenia grade 4 for >7 days’ duration.

*Note*: In the event of a grade 4 neutropenia, a full blood count was performed no more than 7 days after the onset of the event to determine if a DLT occurred. The patient was monitored closely until resolution to grade 3 or less.

- Febrile neutropenia (fever of unknown origin without clinically or microbiologically documented infection).
- Infection (documented clinically or microbiologically) with grade 3 or 4 neutropenia (absolute neutrophil count <1.0 × 10^9^/L).
- Thrombocytopenia grade 3: associated with clinically significant bleeding; requiring platelet transfusion.
- Thrombocytopenia grade 4.
- Grade 3 or 4 toxicity to organs other than the bone marrow including grade 3 and 4 biochemical adverse events (AEs) and DLTs, excluding the following: grade 3 nausea; grade 3 vomiting in patients who had not received optimal treatment with anti-emetics; grade 3 diarrhoea in patients who had not received optimal treatment with anti-diarrhoeal; any grade 3 elevation of AST, ALT, alkaline phosphatase (ALP) (of liver origin), or gamma-glutamyl transferase (GGT) lasting ≤7 days. Elevations in ALP were confirmed to be of liver origin by fractionation of ALP subsets.

*Note*: In the event of a grade 3 or higher elevation in ALT or AST, follow-up laboratory assessments were performed every 48 to 72 hours until reduced to grade 2 or less. In April 2015, the DLT definition was modified to exclude transient grade 3 liver function test elevations, based on the review of the available safety data from part A and feedback from the regulatory agency.

- A concurrent elevation of ALT >3 × the ULN and total bilirubin >2 × ULN in a patient in whom there was no evidence of biliary obstruction or other causes that could reasonably explain the concurrent elevation.
- Death due to drug-related complications.
- Cardiac: QTc prolongation (any QTc interval ≥500 msec or any change in QTc interval ≥60 msec from baseline) on ECG, unless related to an electrolyte abnormality and prolongation resolved with correction of electrolyte abnormality; Any of the following (CTCAE criteria): grade 2 or greater ventricular arrhythmia (second or third degree AV block), severe sustained/symptomatic sinus bradycardia less than 45 beats per minute (bpm) or sinus tachycardia >120 bpm not due to other causes (e.g., fever), persistent supraventricular arrhythmia (e.g., uncontrolled/new atrial fibrillation, flutter, atrioventricular nodal tachycardia, etc.) lasting more than 24 hours, ventricular tachycardia defined as >9 beats in a row or any length of torsades de pointes (polymorphic ventricular tachycardia with long QTc), or unexplained recurrent syncope; Symptoms suggestive of congestive heart failure with confirmed EF <40% (by 2D-echocardiogram or Multiple Gated Acquisition [MUGA] scan) or a relative decrease >20% from historical assessment of EF performed within 12 months; Troponin T: level that was consistent with myocardial infarction.
- Acute hypersensitivity has been recognised as an idiosyncratic reaction without clear relationship to dose of berzosertib occurring in <5% of patients on second infusion of berzosertib, and was not considered a DLT, regardless of grade or need for interruption or discontinuation.
- Any drug-related toxicity that caused interruption of treatment for >2 weeks (14 successive days). If a patient was deemed fit to restart treatment on Day 15 then this was not a DLT.

*Note*: If any change was made to the grade or causality of an AE during the study that could alter its DLT status, the sponsor was informed immediately as this could affect dose escalation decisions.

# Supplementary Table S1. Number of patients experiencing any treatment-related AE of grades 3–4 following treatment with berzosertib + gemcitabine or berzosertib + gemcitabine + cisplatin, stratified by dosing cohort (combination safety set).

| **Dose Level** | **Number of patients with treatment-related AEs of grades 3–4^a^ (n, %)** |
| --- | --- |
| ***Berzosertib + gemcitabine*** | |
| Berzosertib 36 mg/m^2^ + gemcitabine 875 mg/m^2^ | 1 (33.3) |
| Berzosertib 60 mg/m^2^ + gemcitabine 875 mg/m^2^ | 1 (25.0) |
| Berzosertib 72 mg/m^2^ + gemcitabine 875 mg/m^2^ | 2 (28.6) |
| Berzosertib 90 mg/m^2^ + gemcitabine 500 mg/m^2^ | 4 (66.7) |
| Berzosertib 140 mg/m^2^ + gemcitabine 500 mg/m^2^ | 3 (37.5) |
| Berzosertib 210 mg/m^2^ + gemcitabine 500 mg/m^2^ | 2 (66.7) |
| Berzosertib 210 mg/m^2^ + gemcitabine 750 mg/m^2^ | 3 (100.0) |
| Berzosertib 210 mg/m^2^ + gemcitabine 875 mg/m^2^ | 6 (85.7) |
| Berzosertib 210 mg/m^2^ + gemcitabine 1000 mg/m^2^ | 4 (66.7) |
| Total | 26 (52.0) |
| ***Berzosertib + gemcitabine + cisplatin*** | |
| Berzosertib 90 mg/m^2^ + gemcitabine 875 mg/m^2^ + cisplatin 60 mg/m^2^ | 6 (100.0) |
| Berzosertib 120 mg/m^2^ + gemcitabine 875 mg/m^2^ + cisplatin 60 mg/m^2^ | 2 (100.0) |
| Total | 8 (100.0) |

^a^No patients experienced any treatment-related AEs of grade 5.

# Supplementary Table S2. Pharmacokinetic parameters of berzosertib in plasma after single-ascending doses of berzosertib in the lead-in period and after administration of gemcitabine or gemcitabine + cisplatin in cycle 1 (PK analysis set). Data reported as mean (% coefficient of variation)

| **Berzosertib dose (mg/m^2^)** | ***n*** | **C_max_ (ng/mL)** | **AUC_0–∞_**  **(ng∙h/mL)** | **V_ss_ (L)** | **CL (L/h)** | **t_½_ (h)** |
| --- | --- | --- | --- | --- | --- | --- |
| **Lead-in period – berzosertib** **alone** | | | | | | |
| 18 | 3 | 72.0 (28) | 351 (14) | 1600 (22) | 98.2 (13) | 14.0 (24) |
| 36 | 3 | 176 (22) | 913 (46) | 1280 (16) | 90.2 (57) | 14.2 (27) |
| 60 | 4 | 345 (44) | 1700 (32) | 1280 (44) | 77.8 (50) | 15.0 (18) |
| 72 | 7 | 536 (26) | 2280 (22) | 1040 (35) | 62.5 (31) | 15.2 (20) |
| 90 | 6 | 451 (27) | 2370 (27) | 1320 (36) | 74.5 (28) | 17.2 (20) |
| 140 | 5 | 817 (26) | 4550 (26) | 1150 (25) | 60.6 (25) | 17 (15) |
| 210 | 2 | 808 (39) | 6130 (28) | 1550 (46) | 76.3 (29) | 16.8 (22) |
| **Cycle 1 – berzosertib + gemcitabine** | | | | | | |
| 210 | 16 | 899 (41) | 6690 (31) | 1250 (22) | 62.3 (31) | 17.3 (24) |
| **Cycle 1 – berzosertib + gemcitabine + cisplatin** | | | | | | |
| 90 | 6 | 571 (34) | 3100 (39) | 983 (35) | 64.2 (45) | 14.2 (27) |
| 120 | 2 | 486 (28) | 4160 (3) | 1240 (14) | 55.5 (1) | 17.6 (14) |

Abbreviations: AUC_0-∞_, area under the concentration versus time curve from the time of dosing extrapolated to infinity; CL, clearance; C_max_, maximum observed concentration; t_½_, terminal phase half-life; V_ss_, volume of distribution at steady-state.
